# Evaluation of Emotional Intelligence among Master’s Degree Students in Nursing and Midwifery: A Cross-Sectional Survey

**DOI:** 10.3390/ijerph17176347

**Published:** 2020-08-31

**Authors:** Fabiana Cassano, Andrea Tamburrano, Claudia Mellucci, Caterina Galletti, Gianfranco Damiani, Patrizia Laurenti

**Affiliations:** 1 Section of Hygiene, Woman and Child Health and Public Health, Università Cattolica del Sacro Cuore, 00168 Roma, Italy; fabiana.cassano94@gmail.com (F.C.); claudia.mellucci@gmail.com (C.M.); caterina.galletti@unicatt.it (C.G.); gianfranco.damiani@unicatt.it (G.D.); patrizia.laurenti@unicatt.it (P.L.); 2 Woman and Child Health and Public Health, Fondazione Policlinico Universitario A. Gemelli IRCCS, 00168 Roma, Italy

**Keywords:** emotional intelligence, nursing, medical education, midwifery, students, survey, EIS questionnaire

## Abstract

Emotional intelligence is an important skill for nurses and midwives and leads them to cleverly work in various fields and contexts, successfully handling colleagues, patients and their families. The aim of this cross-sectional study is to evaluate the relationship between emotional intelligence, sociodemographic and academic variables in current and former master’s degree students in nursing and midwifery, through the administration of a questionnaire to 71 subjects. Emotional intelligence is significantly related to gender. Females showed higher scores (0.2 points higher than men) for emotional intelligence factors, highlighting an excellent ability to “evaluate and express emotions in relation to others”. Moreover, significant differences in academic performances are shown: both females and midwives demonstrated higher academic performance (a mean degree mark 3.8 points higher than men and a mean degree mark 2.6 point than nurses, respectively). High levels of emotional intelligence in individuals who carried out training activities in the organization area are also evident: These subjects have an ability regarding the “regulation of emotion in the others”, which is significantly higher (*p* = 0.01) than those interested in other master’s degree areas. Emotional intelligence (EI) is strongly linked to the individual’s characteristics and their personalities and differs from technical and professional skills. EI is also an excellent predictor of professional success.

## 1. Introduction

The discovery of emotional intelligence has generated increasing interest during the past decade. Emotional intelligence (EI) is defined by Salovey and Mayer [1] as “the subset of social intelligence that involves the ability to monitor one’s own and others’ feelings and emotions, to discriminate among them and to use this information to guide one’s thinking and action”. According to Goleman, this is an essential skill for the emotional life, relationships, work and social activities of most, if not all, people [2]. Competency in understanding one’s own and others’ emotions consists of knowing the causes and consequences of different emotions as well as being able to differentiate between varying emotions. Good EI levels allow to harness emotions to guide cognitive activities and solve problems, for example, by drawing on positive moods to enhance creative thoughts. Managing one’s own and others’ emotions consists of elaborating emotions, heading situations and achieving individual goals.

In 1996, Goleman defined EI as “Being able to motivate oneself and persist in the face of frustrations; to control impulse and delay gratification; to regulate one’s moods and keep distress from swamping the ability to think; to empathize and to hope” [3]. It is important to highlight the relevance of the power of emotions and relationships in the work environment, and to study the deep impact of EI.

A large number of research studies underline the need to increase EI in the following health care areas:

(1) Education and academic performance: Several studies have shown that the EI has a significant correlation with academic success [4,5,6,7,8]. EI is an academic and clinical performance’s indicator. Moreover, some studies, including Schenk and Harper [8] and Rankin [7], have shown that low levels of EI are correlated with a higher rate of attrition from nursing degree courses. The students who received emotional intelligence training scored significantly higher and were more likely to complete their first year of university than the other students who received no intervention [9].
(2) Quality of care: An important discovery for nursing care was reported by Ezzatabadi et al. [10], stating that nurse’s EI has an influence on the quality of nursing care and job satisfaction. Study results showed that emotional intelligence is directly related to job satisfaction and affects the quality of assistance. Moreover, when health care personnel recognize EI as the mainstay of success in personal and professional life, patient satisfaction increases [11].
(3) Nursing leadership: High levels of emotional intelligence in nurse leaders are linked to high performances in the work environment, inspiring, motivating and stimulating the staff who work with them [12,13]. Prati et al. [14] proposed that EI is “essential to effective team interaction and productivity” and “Emotional Intelligence of the team leader is important to the effective functioning of the team. The leader serves as a motivator to accomplish collaboration and supportive relationships among team members. The emotionally intelligent team leader also provides a transformational influence over the team”.
(4) Sociodemographic variables: Por et al. [15] showed that EI increases with age and education. Snowden et al. [16] also reported that EI increases with age and has a greater representation among women. According to [17,18,19], people with high levels of emotional intelligence can effectively manage positive and negative emotions. They also manage stress by facing emotional challenges in the clinical context, effectively integrating and managing conflicts [20]. Moreover, Harter [21] stressed the relationship between demographic variables and work, showing how some variables could manage different stressors.

## 2. Material and Methods

### 2.1. Aim

The aim of the current study is to evaluate the relationship between EI, sociodemographic and academic variables, in current and former nurses’ and midwives’ students, and to discuss the value of EI as a predictor of professional success.

### 2.2. Design

A cross-sectional study was performed using a 33-item validated questionnaire (Supplementary Materials).

### 2.3. Participants

The sample comprised a total of 71 current and former students from four academic years of a master’s degree course in Nursing and Midwifery in a large University Hospital in Rome (Italy). The course aimed to develop management, care, training and research skills for professional nurses and midwives. Participants were composed of 45 students attending a master’s degree course (1st and 2nd year) and another 26 who had finished their studies in the previous years (former students). In this way it was possible to evaluate emotional intelligence abilities both inside and outside the academic context. A convenience sample method was used.

### 2.4. Data Collection

The present work was based on the administration of the Emotional Intelligence Scale (EIS) developed by Schuttle et al. in 1998 [22], then validated in the Italian language by Grazzani et al. in 2009 [23]. Compared to other questionnaires, the EIS is a helpful scale to measure individual personality traits, in terms of psychometric properties, and is reliable due to its high internal consistency [24]. Ciucchi et al. [25] confirmed the convergent validity of the scale with the measure of emotional self-efficacy. In 2009, Grazzani et al. [23] revealed the test’s multidimensionality using an exploratory factor analysis with rotation on three EIS characters; Cronbach’s alpha was then calculated for emergent factors, in order to verify the internal consistency of each individual dimension. The EIS structure was subjected to a further verification through a confirmatory factor analysis, which confirmed the test validity for adaptation in educational contest.

The first section was added to the questionnaire to collect current and former students’ personal and academic data.

The following section, the EIS questionnaire (Supplementary Materials), consists of 33 items, associated with a five-point Likert scale (from “strongly disagree” to “strongly agree”).

Grazzani et al. [23] identified and measured 3 factors of emotional intelligence through this questionnaire:

- Factor 1: regulation of emotion in others. It includes items related to the ability to recognize other people’s emotions through their facial expression and tone of voice (5, 18, 25, 29, 32, 33). In addition, items 26 and 30 are related to the ability to feel empathy for others. Items 1 and 4 are linked to verbal communication ability.
- Factor 2: regulation of emotion in the self. This includes items related to the ability to recognize one’s emotions and the situations associated to them (9, 19, 22). In addition, item 21 investigates the ability to control one’s emotions.
- Factor 3: utilization of emotion. It includes items related to the ability to monitor one’s mood (3, 10), and to evaluate and act on mood to change it (14) or to use it to solve problems (17, 20, 27, 31). In addition, items 16 and 24 investigate the ability to regulate emotions in others, determining positive feelings towards oneself.

Based on the results obtained with the factor analysis conducted by Grazzani et al. [23] on the Italian version of the EIS Questionnaire, items with saturations lower than 0.30 and ambiguous items (with saturations > 0.30 on more factors) have also been eliminated in this study.

The link to the questionnaire was sent to participants’ e-mail addresses and the survey was filled on a web-based platform. The data were anonymously and automatically collected in an electronic database, and handled by the researchers only. No information on the responder’s identity was requested. Data collection occurred between April and September 2019.

### 2.5. Data Analysis

All the answers were collected within an anonymous database. For the analysis of the answers, according to the factor analysis conducted by Grazzani et al. [23] and confirmed by Ciucci et al. [25], each question was weighted according to the factor to which it refers. The weights of each question are reported in Table 1.

In the first section (concerning the sociodemographic characteristics, training, professional qualification), a descriptive analysis was conducted, reporting answers’ frequency, percentage, mean and standard deviation.

Furthermore, the three dimensions of emotional intelligence were evaluated in relation to sociodemographic parameters, the respondent’s education and their professional qualification. For quantitative variables, following a normal distribution, a Student’s *t*-test and analysis of variance (ANOVA) were used; for qualitative variables Yates’s chi-squared test was used. Finally, the relationship between emotional intelligence, age and academic performance was investigated through the Pearson’s correlation coefficient (*r*). The level of significance was set at 0.05. A statistical analysis was conducted with STATA software ver.13.1 (Statacorp, College Station, TX, USA).

### 2.6. Ethical Considerations

Current/former nurses’ and midwives’ students were informed by the Nursing Services Administration of the University about the objective of the survey and their participation was voluntary and confidential. They were invited to participate without any obligations. The data were collected and analyzed anonymously.

Ethical approval for this study was obtained from the School of Science in Nursing and Midwifery of the Catholic University of Rome.

The study is compliant with the Helsinki Declaration and EU Regulation 2016/679 (GDPR) concerning the processing of personal data.

### 2.7. Validity and Reliability

For this study, the administration of the EIS questionnaire and the data analysis were strictly conducted according to Grazzani et al.’s methodology [22,23]. Due to the small sample size, this study was entirely based on the results of the exploratory and confirmatory factor analysis from the EIS questionnaire, carried out by Grazzani et al. [23], as stated in the “Data Collection” section.

## 3. Results

### 3.1. Sample Characteristics

A total of 71 out of 105 current and former students, to which the questionnaire was sent, completed the survey (67.7% response rate). The sample is composed of 18 students attending the first year of the course (25.4%), 27 students in the second year (38%) and 26 former students (36.6%). 57 of these are females (80.3%) and 14 are males (19.7%). The mean age is 31.7 (from 22 to 59—SD 9.2) years. A total of 62 of them are nurses (87.3%) and 9 are midwives (12.7%).

In total, 22 current/former students (31.0%) carried out training activities in the organization/management area, 21 (29.6%) in nursing and midwifery care, 17 (23.9%) in the area of training and education, while 11 (15.5%) attended the research area. Further information on sample’s characteristics are given in Table 2, Table 3 and Table 4.

Significant differences on academic performance regarding both sample’s gender and professional qualification were observed.

Considering the mean degree mark, females had a score of 108.4 (SD 4.5), males a score of 104.6 (SD 8.1). This difference, equal to 3.8 points, is statistically significant (*p* = 0.02, Student’s *t*-test). Nurses registered a score of 107.2 (SD 5.9), midwives of 109.8 (SD 3.3). This difference is equal to 2.6 points (*p* = 0.07, Student’s *t*-test).

Considering the mean exam mark, females had a score of 27.5 (SD 1.4), males a score of 26.5 (SD 1.9). This difference is equal to 1.0 points (*p* = 0.08, Student’s *t*-test). Nurses registered a score of 27.1 (SD 1.5), midwives of 28.8 (SD 0.8). This difference, equal to 1.7 points, is statistically significant (*p* < 0.01, Student’s *t*-test).

### 3.2. EI, Gender and Age

**Figure 1:**
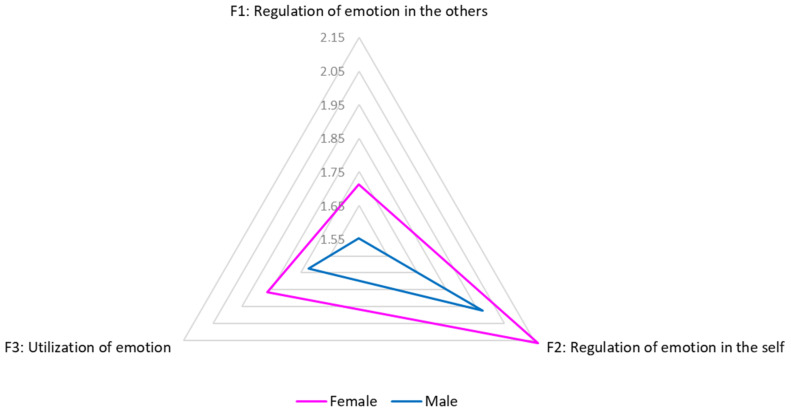
Graphical representation of emotional intelligence factors by gender. Considering age, no significative correlations were found with emotional intelligence (EI) factors (Pearson’s *r*; *p* > 0.05).

Analyzing the sample’s gender, in all EI factors women showed a mean score of 0.2 points higher than men. This difference is statistically significant only for Factor 1 (*p* = 0.03, Student’s *t*-test). This evidence are represented in Table 5 and Figure 1.

### 3.3. EI and Training

The master’s degree program offers four main study areas: nursing and midwifery care, training and education, organization/management, and research.

Considering Factor 1, people who carried out training activities in the organization area showed the highest mean score of 1.8 (SD 0.2). The analysis of the variance also shows statistically significant differences between the training areas (*p* = 0.01, ANOVA).

For Factor 2, people who carried out training activities in the research and the organization areas recorded the highest mean score of 2.2 (SD 0.5).

For Factor 3, people who carried out training activities in the nursing and midwifery care and the organization area showed the highest mean score of 1.9 (SD 0.6 and 0.2, respectively).

Considering academic performance, no significative correlations were found with EI factors.

**Figure 2:**
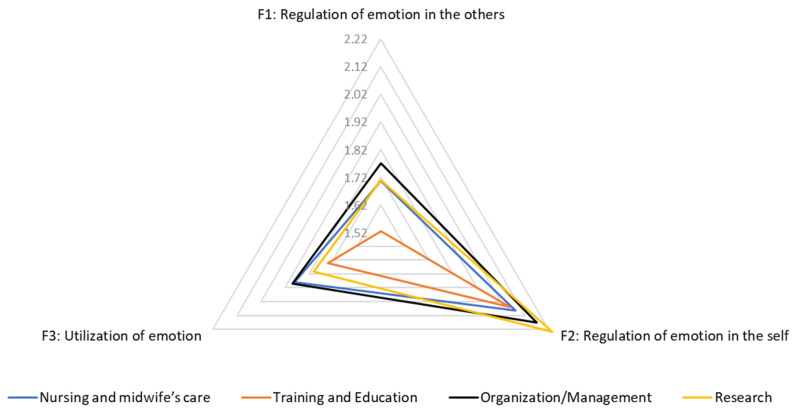
Graphical representation of emotional intelligence factors by training activity.

The results are shown in Table 6 and Table 7 and Figure 2.

### 3.4. Other Considerations

Finally, other correlations are worthy of mention.

Females registered a mean degree mark 3.8 points higher than males (*p* = 0.02, Student’s *t*-test), and a mean exam mark 1.0 points higher than men (*p* = 0.08, Student’s *t*-test). Data on midwives’ academic performance are also interesting, because they showed a mean degree mark 2.6 points higher than nurses (*p* = 0.07, Student’s *t*-test), and a mean exam mark 1.7 points higher than nurses (*p* < 0.01, Student’s *t*-test).

## 4. Discussion

### 4.1. Sociodemographic Variables

Females showed higher scores in all EI factors, highlighting a statistical significance only for Factor 1, the “Evaluation and expression of emotions in relation to others”. This result agrees with Snowden et al.’s study [16], who reported that women have a higher level of EI than men. Many studies report that women tend to demonstrate greater compassion and empathy, greater abilities in social and emotional intelligence, and greater doubt about feelings and decisions than men; the literature is, however, mixed in terms of differences on self-awareness and self-control [26,27].

Age influences emotional maturity. Por et al. [15] identified a positive relationship between nursing student’s age (*r*_s_ = 0.18; *p* < 0.05) and their EI. Snowden et al. [16] also reported that EI increases with age. Unlike these studies, our results on this topic are uncertain: no strong relationships were found between age and EI Factors.

In any case, the research literature agrees that an increased age and being female are both associated with significant increases in emotional intelligence. Accordingly, older people are more socially intelligent and better able to manage their feelings and understand others’ feelings and body language as well. Moreover, the research literature on gender has suggested that women tend to have better interpersonal skills than men. [28,29].

In our study, being either a midwife or a nurse does not influence EI levels (Table 7). Por et al. [15] reported that nursing students with the highest educational background demonstrated a high EI (*r*_s_ = 0.23; *p* < 0.01). Contrary to Por et al.’s study [15], our results show that no significant differences exist between those who attended a master/specialization course and those who did not (Table 7). It could be hypothesized that a specialized educational background, which requires longer study than the basic degree, does not influence emotional intelligence, identifying EI as an intrinsic capacity of the individual’s personality.

### 4.2. Academic Performance

Codier and Odell [4] showed a relationship between EI and academic performance among nursing students. Gharetepeh et al. [30] demonstrated that students with high academic performance had a higher level of EI than students with a lower performance. Unlike these studies, we did not find a strong relationship between academic success and EI. This could be a confirmation that EI is an aspect of an individual’s personality, independent from performance. McCrae and John [31] studied the “five-factor model” of personality and showed the relationship between neuroticism, extraversion, openness, agreeableness, conscientiousness and academic success. By far, conscientiousness is the strongest and most consistent predictor of academic success about personality [32]. This positive relationship is generally attributed to an organized, hard-working, and motivated approach to study that more conscientious individuals display.

### 4.3. Training

From the analysis of the relationship between training and emotional intelligence, the participants who trained in organization and management have higher levels of EI. They showed an ability to regulate emotion in others (Factor 1) significantly better than those interested in other areas (*p* = 0.01, ANOVA). This phenomenon means that subjects with a greater emotional intelligence prefer to continue their university education to improve their competences and abilities and to enhance their skills in the field of organization and management. Individuals interested in this field require more self-control and self-awareness to manage work situations, never forgetting essential emotions.

Managers need to know how to work with self and with the other, how manage emotional intelligence to connect the self with external context: whoever wants to be a good leader needs to study to enhance their abilities. In 2016, Başoğul and Özgür’s study [33] showed that most conflicts among nurses occur with their colleagues in the same unit, as they often relate to working conditions and inadequate communication. Skills requiring high level of emotional intelligence, such as problem solving, interpersonal relations, and stress management, play a key role in effective conflict management. There is a need for training programs designed to improve emotional intelligence among nurses in order to manage conflicts among staff, which are an inevitable occurrence in any workplace.

### 4.4. Limitations

The first limit of the study is the small sample size. The number of participants is in accordance with the setting of the master’s degree in nursing and midwifery, where small groups of students can be enrolled. In any one-year, 30/35 students are selected to attend the course. In this academic context, it was not possible to conduct an internal exploratory and confirmatory factor analysis on the questionnaire along the lines of Grazzani et al.’s study.

A larger sample could lead to new and interesting relationships with variables, currently not significant.

The second limit is represented by the setting: this is a monocentric study and data are restricted to one reality. Further studies in other academic contexts could be carried out in order to test thisevidence in more heterogeneous groups of students.

The last study’s limitation is related to the survey, whose items do not investigate specific emotional experiences, as stated by Grazzani et al. [23], and do not consider other work-related effects (i.e., stress, burnout). This gap could be filled by proposing tools that measure specific emotional aspects and the work context (regulation of anger, sadness, guilt, work-related stress, etc.).

## 5. Conclusions

The EIS questionnaire proves to be an easy instrument to use and to interpret, suitable for measuring people’s EI in an effective way.

The study represents a step forward for the nursing and midwifery courses, showing that the subjective sphere is much related to EI than the objective one. EI is strongly linked to the individual’s characteristics and their personality (soft skills) which differs from technical and professional skills (hard skills). Psychologist Daniel Goleman [2] breaks down emotional intelligence skills into five basic parts: self-awareness, self-management, self-motivation, empathy, and social skills. As one young lawyer put it in a recent Huron Consulting white paper, “I wasn’t the smartest student in law school ... but I have always understood what makes people tick and always known my own strengths and weaknesses. I’m not afraid to ask questions and not afraid to look dumb”. Knowing what these EI skills and traits are allows one to focus on them and develop greater emotional intelligence [34].

As such, EI is a reliable predictor of success: high EI levels lead to a high work efficacy, quality of life, self-awareness, relationship management and decision making. Cadman and Brewer [35] believed that during a university course’s selection, it was necessary to adopt strategies to determine levels of candidates’ emotional intelligence. Consequently, this aspect should be included in university curricula and in lifelong learning. EI can be applied to all working and university fields to develop professional and managerial skills and train more oriented professionals. Students’ emotional intelligence need to be evaluated first, and then trained, as the EI high level is strictly linked to leadership competence, especially for people who work in the field of healthcare.

Students’ EI capabilities will lead to healthcare professionals being able to work in various fields and contexts, working successfully with colleagues, patients and their families. Rosenthal [36] discovered that people who were good at identifying others’ emotions had more success in their work as well as in their social lives. Thus, empathy is particularly important in contributing to occupational success.

## Figures and Tables

**Table 1:** Factor saturations and correlations between emotional intelligence factors (adapted from Grazzani et al., 2009 with permission).

Item	Factor 1	Factor 2	Factor 3
1	0.43		
4	0.36		
5	0.27		
15	0.39		
18	0.60		
25	0.55		
26	0.47		
29	0.56		
30	0.52		
32	0.42		
33	0.28		
9		0.60	
19		0.58	
21		0.42	
22		0.74	
3			0.33
10			0.34
14			0.45
16			0.54
17			0.74
20			0.60
24			0.35
27			0.38
31			0.42
correlations			
factor 1	1.00	-	-
factor 2	0.43	1.00	-
factor 3	0.62	0.35	1.00

**Table 2:** Frequency distribution (values and row percentages) and *p*-value (Yates’s chi-squared test) of sample’s working situation, stratified by year of course, gender and professional qualification.

**Year of Course**	**Employed**	B	**Total**	***p*-Value**
1st year	10 (55.5%)	8 (44.4%)	18 (100%)	<0.01
2nd year	23 (85.1%)	4 (14.8%)	27 (100%)	
Former student	25 (95.1%)	1 (3.9%)	26 (100%)	
Total	58 (81.7%)	13 (18.3%)	71 (100%)	
**Gender**	**Employed**	**Unemployed**	**Total**	***p*-Value**
Female	45 (78.9%)	12 (21.1%)	57 (100%)	0.41
Male	13 (92.9%)	1 (7.1%)	14 (100%)	
**Qualification**	**Employed**	**Unemployed**	**Total**	***p*-Value**
Nurse	51 (82.3%)	11 (17.7%)	62 (100%)	0.89
Midwife	7 (77.8%)	2 (22.2%)	9 (100%)	

**Table 3:** Frequency distributions (values and row percentages) and *p*-value (Yates’s chi-squared test) of sample’s postgraduate training, stratified by year of course, gender and professional qualification.

**Year of Course**	**Masters or Specialization Courses**	**No Title**	**Total**	***p*-Value**
1st year	10 (14.1%)	8 (11.3%)	18 (100%)	0.34
2nd year	15 (21.1%)	12 (16.9%)	27 (100%)	
Former student	19 (26.7%)	7 (9.8%)	26 (100%)	
Total	44 (61.9%)	27 (38.1%)	71 (100%)	
**Gender**	**Masters or Specialization Courses**	**No Title**	**Total**	***p*-Value**
Female	33 (57.9%)	24 (42.1%)	57 (100%)	0.26
Male	11 (78.6%)	3 (21.4%)	14 (100%)	
**Qualification**	**Masters or Specialization Courses**	**No Title**	**Total**	***p*-Value**
Nurse	38 (61.3%)	24 (38.7%)	62 (100%)	0.95
Midwife	6 (66.7%)	3 (33.3%)	9 (100%)	

**Table 4:** Mean, standard deviation and *p*-value (Student’s *t*-test and ANOVA) of sample’s exam and degree marks, stratified by year of course, gender and professional qualification.

**Year of Course**	**Exam Mark**	**Degree Mark**	**Total**	***p*-Value Exam Mark**	***p*-Value Degree Mark**
1st year	27.4 (SD 2.3)	106.9 (SD 5.7)	18	0.89	0.78
2nd year	27.4 (SD 1.1)	107.4 (SD 5.4)	27		
Former student	27.2 (SD 1.7)	108.1 (SD 6.0)	26		
Total	27.3 (SD 1.5)	107.5 (SD 5.7)	71		
**Gender**	**Exam Mark**	**Degree Mark**	**Total**	***p*-Value Exam Mark**	***p*-Value Degree Mark**
Female	27.5 (SD 1.4)	108.4 (SD 4.5)	57	0.08	0.02
Male	26.5 (SD 1.9)	104.6 (SD 8.1)	14		
**Qualification**	**Exam Mark**	**Degree Mark**	**Total**	***p*-Value Exam Mark**	***p*-Value Degree Mark**
Nurse	27.1 (SD 1.5)	107.2 (SD 5.9)	62	<0.01	0.07
Midwife	28.8 (SD 0.8)	109.8 (SD 3.3)	9		

**Table 5:** Mean, standard deviation and *p*-value (Student’s *t*-test) of sample’s EI factors score, stratified by gender.

Emotional Intelligence	Female (N = 57)	Male (N = 14)	Total (N = 71)	*p*-Value
Factor 1	1.7 (SD 0.2)	1.5 (SD 0.3)	1.7 (SD 0.2)	0.03
Factor 2	2.2 (SD 0.4)	2.0 (SD 0.4)	2.1 (SD 0.4)	0.13
Factor 3	1.9 (SD 0.3)	1.7 (SD 0.4)	1.8 (SD 0.3)	0.09

**Table 6:** Mean, standard deviation and *p*-value (ANOVA) of sample’s EI factors score, stratified by training activities.

Emotional Intelligence	Nursing and Midwifery Care (N = 21)	Training and Education	Organization/Management	Research	Total (N = 71)	*p*-Value
		(N = 17)	(N = 22)	(N = 11)		
Factor 1	1.7 (SD 0.1)	1.5 (SD 0.3)	1.8 (SD 0.2)	1.7 (SD 0.3)	1.9 (SD 0.2)	0.01
Factor 2	2.1 (SD 0.4)	2.1 (SD 0.5)	2.2 (SD 0.5)	2.2 (SD 0.5)	2.1 (SD 0.4)	0.64
Factor 3	1.9 (SD 0.6)	1.7 (SD 0.5)	1.9 (SD 0.2)	1.8 (SD 0.3)	1.8 (SD 0.3)	0.40

**Table 7:** Mean, standard deviation and *p*-value (Student’s *t*-test) of sample’s EI factors score, stratified by professional qualification and postgraduate training.

Emotional Intelligence	Nurse (N = 62)	Midwife (N = 9)	*p*-Value	Masters or Specialization Courses (N = 44)	No Title (N = 27)	*p*-Value
Factor 1	1.7 (SD 0.3)	1.7 (SD 0.1)	>0.99	1.7 (SD 0.3)	1.7 (SD 0.1)	>0.99
Factor 2	2.1 (SD 0.4)	2.1 (SD 0.4)	>0.99	2.1 (SD 0.4)	2.2 (SD 0.4)	0.3
Factor 3	1.8 (SD 0.3)	1.9 (SD 0.2)	0.2	1.8 (SD 0.4)	1.9 (SD 0.2)	0.2

## Supplementary Materials

The following are available online at https://www.mdpi.com/1660-4601/17/17/6347/s1, EIS Questionnaire (English translation), Università Cattolica del Sacro Cuore di Roma.

## References

[1] Salovey P., Mayer J.D. (1990). Emotional Intelligence. Imagination, cognition and personality. Sage J..

[2] Goleman D. (1995). Emotional Intelligence.

[3] Goleman D. (1996). Emotional Intelligence: Why It Can Matter More than IQ.

[4] Codier E., Odell E. (2014). Measured EI ability and grade point average in nursing students. Nurs. Educ. Today.

[5] Beauvais A.M., Brady N., O’Shea E.R., Griffin M.T.Q. (2011). Emotional Intelligence and nursing performance among nursing students. Nurs. Educ. Today.

[6] Fernandez R., Salamonson Y., Griffiths R. (2012). Emotional Intelligence as a predictor of academic performance in first year accelerated graduate entry nursing students. J. Clin. Nurs..

[7] Rankin B. (2013). Emotional Intelligence: Enhancing values-based practice and compassionate care in nursing. J. Adv. Nurs..

[8] Schenk J.J., Harper M.G. (2013). Emotional Intelligence: An admission criterion alternative to cumulative grade point averages for prelicensure students. Nurs. Educ. Today.

[9] Schutte N.S., Malouff J.M. (2002). Incorporating emotional skills in a college transition course enhances student retention. J. First-Year Exp. Stud. Transit..

[10] Ezzatabadi M.R., Bahrami M.A., Hadizadeh F., Arab M., Nasiri S., Amiresmaili M., Tehrani G.A. (2012). Nurses’ emotional impact on the quality of hospital services. Iran. Red Cresent Med. J..

[11] Akerjordet K., Severinsson E. (2007). Emotional Intelligence: A review of the literature with specific focus on empirical and epistemological perspectives. J. Clin. Nurs..

[12] Smith K.B., Profetto-McGrath J., Cummings G.G. (2009). Emotional Intelligence and nursing: An integrative literature review. Int. J. Nurs. Stud..

[13] Heckemann B., Schols J.M.G.A., Halfens R.J.G. (2015). A reflective framework to foster emotionally intelligent leadership in nursing. J. Nurs. Manag..

[14] Prati L.M., Douglas C., Ferris G.R., Ammeter A.P., Buckley M.R. (2003). Emotional Intelligence, leadership effectiveness, and team outcomes. Int. J. Organ. Anal..

[15] Por J., Barriball L., Fitzpatrick J., Roberts J. (2011). Emotional Intelligence: Its relationship to stress, coping, well-being and professional. Nurs. Educ. Today.

[16] Snowden A., Stenhouse R., Young J., Carver H., Carver F., Brown N. (2015). The relationship between Emotional Intelligence, previous caring experience and mindfulness in student nurses and midwives: A cross sectional analysis. Nurs. Educ. Today.

[17] Ekermans G.G., Brand T. (2012). Emotional Intelligence as a moderator in the stress-burnout relationship: A questionnaire study on nurses. J. Clin. Nurs..

[18] Karimi L., Leggat S.G., Donohue L., Farrell G., Couper G.E. (2013). Emotional rescue: The role of Emotional Intelligence and emotional labour on wellbeing and job stress among community nurses. J. Adv. Nurs..

[19] Zhang P., Li C.Z., Xing F.M., Chen C.X., Tian X.F., Tang Q.Q. (2015). The mediating role of Emotional Intelligence between negative life events and psychological distress among nursing students: A cross sectional study. Nurs. Educ. Today.

[20] Chan J.C.Y., Sit E.N.M., Lau W.M. (2014). Conflict management styles, Emotional Intelligence and implicit theories of personality of nursing students: A cross-sectional study. Nurs. Educ. Today.

[21] Harter S., Greca A.M. (1990). Issues in the assessment of the self-concept of children and adolescents. Through the Eyes of the Child: Obtaining Self-Reports from Children and Adolescents.

[22] Schutte S.N., Malouff J.M., Hall L.H., Haggerty D.J., Cooper J.T., Golden C.J., Dornheim L. (1998). Development and validation of a measure of Emotional Intelligence. Personal. Individ. Differ..

[23] Grazzani I., Antoniotti C., Ciucci E., Menesini E., Primi C. (2009). La misurazione dell’intelligenza emotiva: Un contributo alla validazione italiana dell’Emotional Intelligence Scale (EIS) con adolescenti. G. Ital. Psicol..

[24] Machia V.M., Agnew C.R., Arriaga X.B. (2020). Interdependence, Interaction, and Close Relationship.

[25] Ciucci E., Menesini E., Primi C., Grazzani I., Antoniotti C. (2009). Studio sulle proprietà psicometriche dell’Emotional Intelligence Scale con preadolescenti. Counseling.

[26] Bernet M. Emotional Intelligence: Components and Correlates. https://files.eric.ed.gov/fulltext/ED408535.pdf.

[27] Sutarso T., Bagget L.K., Sutarso P., Tapia M. Effect of Gender and GPA on Emotional Intelligence. https://files.eric.ed.gov/fulltext/ED406410.pdf.

[28] Tannen D. (1990). You just Don’t Understand: Women and Men in Conversation.

[29] Wood J. (2009). Communication, gender, and culture. Gendered Lives.

[30] Gharetepeh A., Safari Y., Pashaei T., Razaei M., Kajbaf M.B. (2015). Emotional Intelligence as a predictor of self-efficacy among students with different levels of academic achievement at Kermanshah University of Medical Sciences. J. Adv. Med. Educ. Prof..

[31] McCrae R.R., John O.P. (1992). An introduction to the 5-factor model and its applications. J. Personal..

[32] Busato V.V., Prins F.J., Elshout J.J., Hamaker C. (2000). Intellectual ability, learning style, personality, achievement motivation, and academic success of psychology students in higher education. Personal. Individ. Differ..

[33] Başoğul C., Özgür G. (2016). Role of Emotional Intelligence in conflict management strategies of nurses. Asian Nurs. Res..

[34] Middleburgh J., Butterworth L. Emotional Intelligence: What Can Learned Lawyers Learn from the Less Learned? The Barrister. http://www.barristermagazine.com/barrister/index.php?id=480.

[35] Cadman C., Brewer J. (2001). Emotional Intelligence: A vital prerequisite for recruitment in nursing. J. Nurs. Manag..

[36] Rosenthal R., McReynolds P. (1977). The PONS test: Measuring sensitivity to nonverbal cues. Advances in Psychological Assessment.

