# Supplementary material for: Evaluation of Emotional Intelligence among Master’s Degree Students in Nursing and Midwifery: A Cross-Sectional Survey
Source: Int J Environ Res Public Health. 2020 Aug 31;17(17):6347. doi: 10.3390/ijerph17176347 (PMC7504047; doi:10.3390/ijerph17176347)
Supplement: Supplementary file 1 [file ijerph-17-06347-s001.pdf]

EIS Questionnaire (English translation)

Università Cattolica del Sacro Cuore di Roma[1]

Dear Colleague,

The aim of this study is to evaluate the Emotional Intelligence (IE) in students and ex-students of the master's degree in Nursing and Midwifery at the *Catholic University of the Sacred Heart of Rome.*

We kindly ask your consent to complete this questionnaire.

We ask you to answer all the questions, there are no right or wrong answers.

Thank you for your time.

EMAIL ADDRESS:

____

[1]Reference: Grazzani, I., Antoniotti, C., Ciucci, E., Menesini, E., and Primi, C. (2009). *La misurazione dell'intelligenza emotiva: un contributo alla validazione italiana dell'Emotional Intelligence Scale (EIS) con adolescenti*. Giornale italiano di psicologia, 36(3), 635-656.

Use of personal data

We inform you that all the information related to participation in this study will be treated in a strictly confidential manner in accordance with the EU Regulation 2016/679 of the European Parliament and of the Council concerning the protection of individuals regarding the processing of personal data, as well as to the free circulation of such data. Pursuant to Law 675/1996 and the subsequent Legislative Decree 196/2003 all the information collected with the questionnaires will be used exclusively for scientific research purposes (art. 12, c. 1, point d). Furthermore, the data collected as part of this investigation are protected by statistical secrecy and therefore cannot be communicated or externalized except in aggregate form, so that no individual reference can be made, and can only be used for purposes statistics (Article 9 of Legislative Decree 6 September 1989, No. 322).

The results of the investigation may be published but its identity and participation will always remain secret. The data collected with the questionnaires will be used exclusively for research purposes indicated above, filed electronically and with the adoption of security measures provided by law.

□ **AGREE** □ **NOT AGREE**

Signature ______________________________ Date ______________

SECTION 1

1.1 ANAGRAPHIC SECTION

1.1.1 GENDER:

- ☐ Male
- ☐ Female

1.1.2 AGE (years):

____

1.1.3 YEAR OF STUDY:

- ☐ 1st
- ☐ 2nd
- ☐ Previous years

1.1.4 INDICATE YOUR PROFESSIONAL ROLE:

- ☐ Nurse
- ☐ Midwife

1.1.5 INDICATE YOUR WORKING SITUATION:

- ☐ Employed
- ☐ Unemployed

1.2 POST-GRADUATE TRAINING

1.2.1 DO YOU HAVE ONE OR MORE OF THE FOLLOWING TITLE?

- ☐ Master
- ☐ Advanced Course

- None

1.2.2 IF YOU HAVE REPORTED "MASTER", INDICATE THE TITLE:

____

1.2.3 IF YOU HAVE REPORTED "ADVANCED COURSE", INDICATE THE TITLE:

____

1.3 ACADEMIC DETAILS

1.3.1 DEGREE MARK (insert vote in numbers or "I don't remember"):

____

1.3.2 MEAN OF EXAM MARK (insert vote in numbers or "I don't remember"):

1.3.3 IN WHICH AREA HAVE YOU DONE YOUR INTERNSHIP?

- Nursing and midwifery care
- Training and education
- Organization / Management
- Research

SECTION 2

The Assessing Emotions Scale

Each of the following items asks you about your emotions or reactions associated with emotions. After deciding whether a statement is generally true for you, use the 5-point scale to respond to the statement. Please circle the "1" if you strongly disagree that this is like you, the "2" if you somewhat disagree that this is like you, "3" if you neither agree nor disagree that this is like you, the "4" if you somewhat agree that this is like you, and the "5" if you strongly agree that this is like you.

There are no right or wrong answers. Please give the response that best describes you.

1 = strongly disagree

2 = somewhat disagree

3 = neither agree nor disagree

4 = somewhat agree

5 = strongly agree

	Item					
1	I know when to speak about my personal problems to others.	1	2	3	4	5
2	When I am faced with obstacles, I remember times I faced similar obstacles and overcame them.	1	2	3	4	5
3	I expect that I will do well on most things I try.	1	2	3	4	5
4	Other people find it easy to confide in me.	1	2	3	4	5
5	I find it hard to understand the non-verbal messages of other people.	1	2	3	4	5
6	Some of the major events of my life have led me to re-evaluate what is important and not important.	1	2	3	4	5
7	When my mood changes, I see new possibilities.	1	2	3	4	5
8	Emotions are one of the things that make my life worth living.	1	2	3	4	5
9	I am aware of my emotions as I experience them.	1	2	3	4	5
10	I expect good things to happen.	1	2	3	4	5
11	I like to share my emotions with others.	1	2	3	4	5
12	When I experience a positive emotion, I know how to make it last.	1	2	3	4	5
13	I arrange events others enjoy.	1	2	3	4	5
14	I seek out activities that make me happy.	1	2	3	4	5
15	I am aware of the non-verbal messages I send to others.	1	2	3	4	5
16	I present myself in a way that makes a good impression on others.	1	2	3	4	5
17	When I am in a positive mood, solving problems is easy for me.	1	2	3	4	5
18	By looking at their facial expressions, I recognize the emotions people are experiencing.	1	2	3	4	5

19	I know why my emotions change.	1	2	3	4	5
20	When I am in a positive mood, I am able to come up with new ideas.	1	2	3	4	5
21	I have control over my emotions.	1	2	3	4	5
22	I easily recognize my emotions as I experience them.	1	2	3	4	5
23	I motivate myself by imagining a good outcome to tasks I take on.	1	2	3	4	5
24	I compliment others when they have done something well.	1	2	3	4	5
25	I am aware of the non-verbal messages other people send.	1	2	3	4	5
26	When another person tells me about an important event in his or her life, I almost feel as though I experienced this event myself.	1	2	3	4	5
27	When I feel a change in emotions, I tend to come up with new ideas.	1	2	3	4	5
28	When I am faced with a challenge, I give up because I believe I will fail.	1	2	3	4	5
29	I know what other people are feeling just by looking at them.	1	2	3	4	5
30	I help other people feel better when they are down.	1	2	3	4	5
31	I use good moods to help myself keep trying in the face of obstacles.	1	2	3	4	5
32	I can tell how people are feeling by listening to the tone of their voice.	1	2	3	4	5
33	It is difficult for me to understand why people feel the way they do.	1	2	3	4	5

THANK YOU FOR YOUR TIME!
